# Exosomes derived from miR-122-modified adipose tissue-derived MSCs increase chemosensitivity of hepatocellular carcinoma

**DOI:** 10.1186/s13045-015-0220-7

**Published:** 2015-10-29

**Authors:** Guohua Lou, Xiuli Song, Fan Yang, Shanshan Wu, Jing Wang, Zhi Chen, Yanning Liu

**Affiliations:** State Key Laboratory for Diagnosis and Treatment of Infectious Diseases, The First Affiliated Hospital, College of Medicine, Zhejiang University, Collaborative Innovation Center for Diagnosis and Treatment of Infectious Diseases, 79# Qingchun Road, 6A-17, Hangzhou, 310003 China; Institute of Genetics, College of Life Science, Zhejiang University, Hangzhou, 310003 China

**Keywords:** Adipose tissue-derived MSC, Exosome, miR-122, Hepatocellular carcinoma, Chemosensitivity

## Abstract

**Background:**

Hepatocellular carcinoma (HCC) displays high resistance to conventional chemotherapy. Considering that microRNA-122 (miR-122) performs an essential function to promote chemosensitivity of HCC cells, an effective vehicle-mediated miR-122 delivery may represent a promising strategy for HCC chemotherapy. An increasing interest is focused on the use of exosomes as biological vehicles for microRNAs (miRNA) transfer. Mesenchymal stem cells (MSCs) are known for their capacity to produce large amounts of exosomes. This study aimed to determine whether adipose tissue-derived MSC (AMSC) exosomes can be used for miR-122 delivery.

**Methods:**

AMSCs were transfected with a miR-122 expression plasmid. At 48 h after transfection, AMSC-derived exosomes (122-Exo) were harvested and added to recipient HCC cells. Expression levels of miR-122 in AMSCs, exosomes, and HCC cells were quantified by real-time PCR. The mRNA and protein levels of miR-122-target genes in recipient HCC cells were quantified by real-time PCR and Western blot, respectively. The effects of 122-Exo on cell viability, apoptosis, and cell cycle of HCC cells were evaluated by MTT and flow cytometry analysis. Xenograft models were used to determine whether 122-Exo can sensitize HCC cells to sorafenib in vivo.

**Results:**

Data showed that miR-122-transfected AMSC can effectively package miR-122 into secreted exosomes, which can mediate miR-122 communication between AMSCs and HCC cells, thereby rendering cancer cells sensitive to chemotherapeutic agents through alteration of miR-122-target gene expression in HCC cells. Moreover, intra-tumor injection of 122-Exo significantly increased the antitumor efficacy of sorafenib on HCC in vivo.

**Conclusions:**

The findings suggest that the export of miR-122 via AMSC exosomes represents a novel strategy to enhance HCC chemosensitivity.

## Background

Most hepatocellular carcinoma (HCC) patients are diagnosed at intermediate advanced stages, during which the only proven therapies are transarterial chemoembolization (TACE) and targeted therapy with the multikinase inhibitor, sorafenib [1]. However, HCC displays high resistance to commonly used chemotherapeutic agents, such as 5-fluorouracil (5-FU) and doxorubicin. Therefore, the discovery of new targets and the development of novel therapeutic approaches to enhance HCC chemosensitivity are urgently needed.

At present, certain progress is being developed in the abovementioned field [2, 3]. Several studies have indicated that non-coding RNA, such as long non-coding RNAs and microRNAs (miRNAs), participate in cancer development and perform important functions in diagnosis and prognosis [4–6]. Moreover, miRNAs are determined to be correlated with chemosensitivity in cancers [7, 8]. The liver-specific microRNA-122 (miR-122) has been found to perform multiple functions in liver physiology and pathology [9]. Notably, the loss or downregulation of miR-122 has been associated with HCC development and progression [10] and is closely related to poor prognosis and metastasis of HCC [11]. Increasing evidence indicates that miR-122 can modulate the chemosensitivity of HCC cells [12]. Ectopic expression of miR-122 in non-expressing HepG2 and Hep3B cells can inhibit tumorigenic properties, such as growth, invasion, and tumor formation in nude mice, as well as can sensitize these cells to doxorubicin and sorafenib [13, 14].

However, a safe and effective vehicle for miR-122 delivery also is a key factor in miR-122-mediated chemotherapy sensitization. Growing interest is focused on the use of exosomes as biological delivery vehicles for miRNA transfer, as exosomes do not elicit acute immune rejection and risk tumor formation [15]. Furthermore, exosomes can be manufactured in culture by incorporating therapeutic miRNAs into exosome-producing cells, thereby possibly enabling personalized treatment [16]. Among the cell types known to produce exosomes, human mesenchymal stem cells (MSCs) are the most prolific producers [17]. Infusion of human MSC-derived exosomes into an immunocompetent mouse model of acute myocardial ischemia has been shown to be therapeutically effective and lacking of evident adverse effects [18].

Adipose tissue-derived MSCs (AMSCs) represent a highly advantageous tool for stem cell-based therapy [19]. The current study investigated whether exosome-mediated transfer of miR-122 via miRNA-modified AMSCs can enhance the chemosensitivity of HCC cells.

## Results

### AMSCs package miR-122 into secreted exosomes

**Fig. 1:**
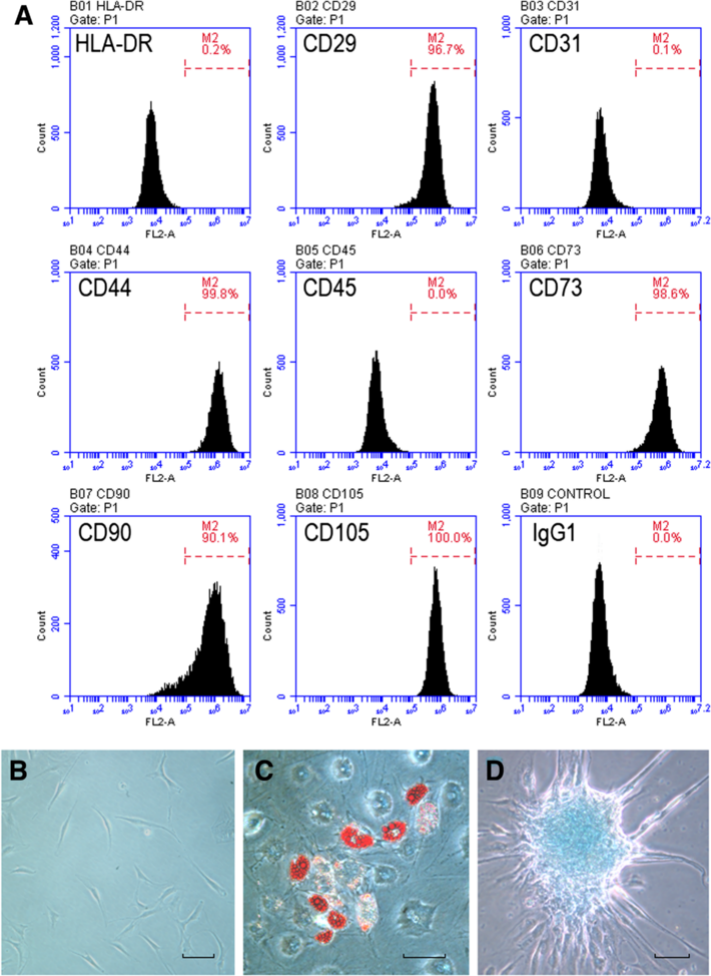
Identification of human adipose tissue-derived mesenchymal stem cells (AMSCs). **a** Flow cytometry analysis of the surface markers in AMSCs. **b** Cellular morphology of AMSCs in culture. **c** Oil Red O staining in AMSCs cultured in adipogenesis differentiation medium for 14 days. **d** Alcian Blue staining in AMSCs cultured in chondrogenesis differentiation medium for 21 days. Scale bar = 50 μm

AMSCs positively expressed CD29, CD44, CD73, CD90, and CD105 but negatively expressed CD31, CD34, CD45, and HLA-DR. Positive staining of Oil Red O or Alcian Blue was observed after adipogenic induction of AMSCs for 14 days or chondrogenic induction for 28 days, respectively (Fig. 1).

**Fig. 2:**
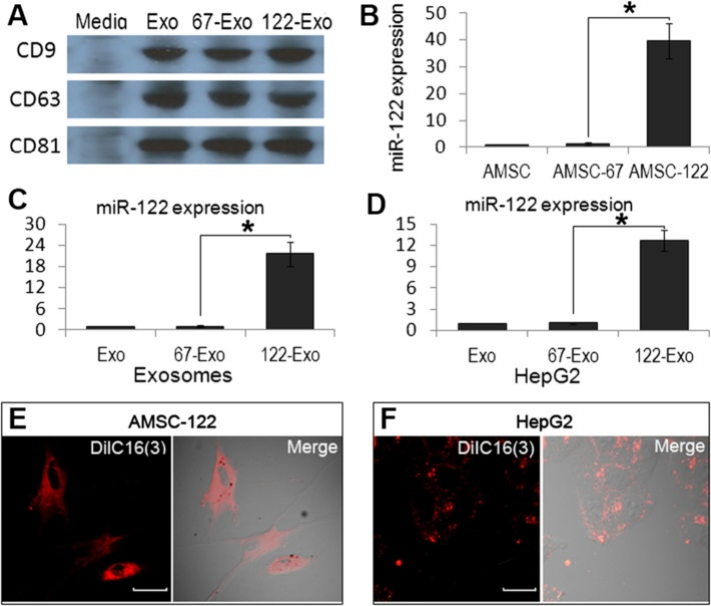
Exosome-mediated miR-122 communication between AMSCs and HepG2 cells. **a** Western blot for CD9, CD63, and CD81 expression in AMSC-derived exosomes. **b**–**d** Real-time PCR detection of miR-122 expression in AMSCs (**b**), AMSC-derived exosomes (**c**), and exosome-treated HepG2 cells (**d**). **e**, **f** Confocal images of AMSC-122 stained with DilC_16_(3), a phospholipid membrane dye. Transfer of fluorescent exosomes from AMSC-122 is apparent in HepG2 cell membranes and cytoplasm. Data are presented as means ± SE. (*p < 0.05, *n* = 3). *AMSC-122* miR-122-transfected AMSC, *AMSC-67* cel-miR-67-transfected AMSC, *Media* precipitates from AMSC-conditioned media, *Exo* naïve AMSC-derived exosomes, *122-Exo* AMSC-122-derived exosomes, *67-Exo* AMSC-67-derived exosomes. Scale bar = 20 μm

AMSCs were transfected with a plasmid encoding for miR-122 or for cel-miR-67 (*Caenorhabditis elegans* miR-67) as control. At 48 h after transfection, extracellular exosomes were isolated from the AMSCs supernatant. AMSC-derived exosomes showed the positive expression of exosomal markers, such as CD9, CD63, and CD81 [17, 20] (Fig. 2a). Afterward, miR-122 expression was measured in AMSCs and exosomes. The expression of miR-122 was 39.7 ± 1.3-fold and 21.6 ± 3.4-fold higher in miR-122-transfected AMSCs (AMSC-122) and their exosomes (122-Exo) than that of miR-122 in cel-miR-67-transfected AMSCs (AMSC-67) and their exosomes (67-Exo), correspondingly (Fig. 2b, c). These data demonstrate that AMSCs can efficiently package plasmid-expressed miR-122 into secreted exosomes.

### Exosomes mediate miR-122 communication between AMSCs and HCC cells

AMSC-122 cells were labeled with a phospholipid membrane dye, DilC_16_ (3), to trace the derived exosomes. After culturing for an additional 48 h, fluorescent exosomes were collected and added to recipient HepG2 cells. Confocal imaging revealed the delivery of labeled exosomes as indicated by the presence of the fluorescent membrane in unlabeled recipient HepG2 cells (Fig. 2e, f). As a further proof, the expression of miR-122 was 10.5 ± 1.4-fold higher in 122-Exo-treated HepG2 cells than that in 67-Exo-treated cells (Fig. 2d). Thus, AMSC-derived exosomes can deliver miR-122 into HCC cells in vitro.

### 122-Exo alters target gene expression in HCC cells

**Fig. 3:**
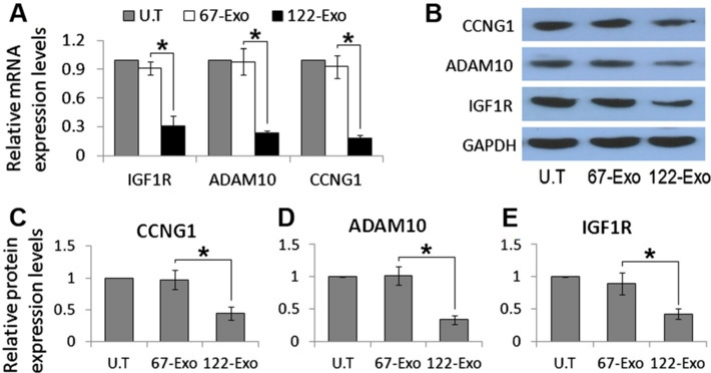
122-Exo alters miR-122-target genes expression in HepG2 cells. **a** mRNA expression levels of miR-122-targeted genes in HepG2 cells treated with exosomes. **b** Western blot analysis of miR-122-targeted genes in HepG2 cells treated with exosomes. **c–e** Relative protein expression levels of CCNG1, ADAM10, and IGF1R in exosome-treated HepG2 cells. Data are presented as means ± SE. (*p < 0.05, *n* = 3). *U.T* untreated, *122-Exo* AMSC-122-derived exosomes, *67-Exo* AMSC-67-derived exosomes

To examine whether 122-Exo mediated-miR-122 communication can alter the expression of miR-122 target genes, such as cyclin G1 (CCNG1), a disintegrin and metalloprotease 10 (ADAM10), and insulin-like growth factor receptor 1 (IGF1R) in hepatoma cells [13, 14], HepG2 cells were exposed to 122-Exo or 67-Exo for 24 h. Both mRNA and protein levels of these genes were downregulated in 122-Exo-treated HepG2 cells in comparison with 67-Exo-treated cells (Fig. 3).

These data suggest that miR-122, which is delivered via AMSC exosomes, is functionally active in acceptor HCC cells. Moreover, 122-Exo may potentially facilitate the sensitivity of HCC cells to chemotherapeutic agents by negative regulation of the expression of miR-122 target genes, which are involved in the drug resistance or sensitivity of cancer cells.

### 122-Exo increases chemosensitivity of HCC cells

**Fig. 4:**
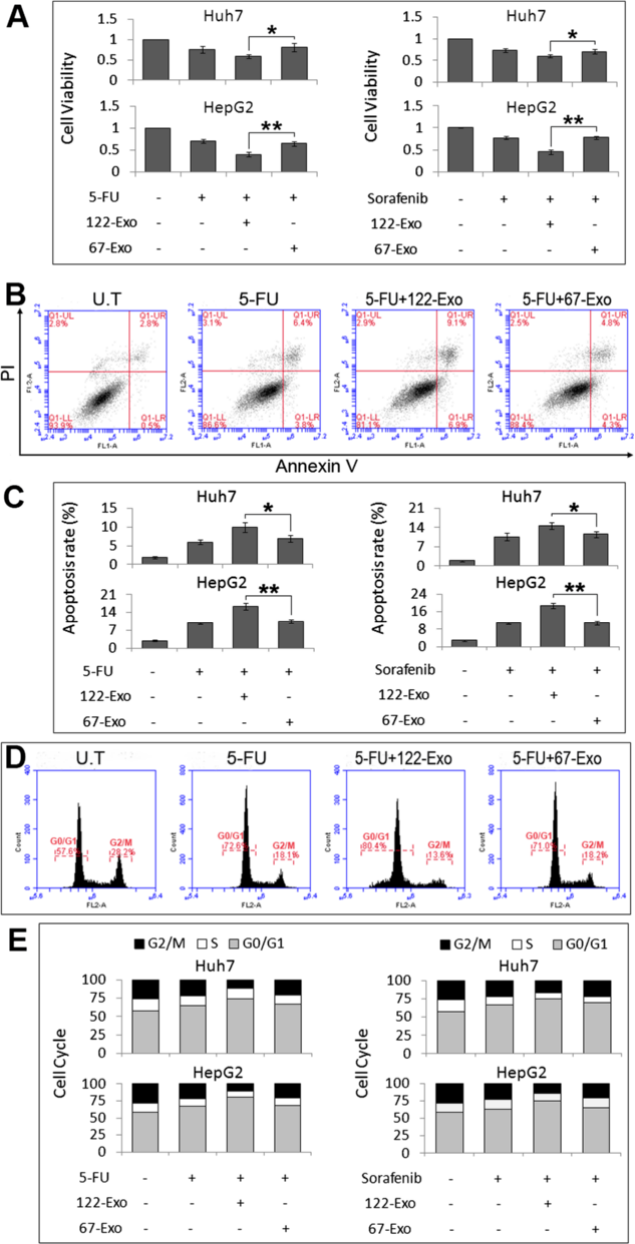
122-Exo sensitizes HCC cells to chemotherapeutic agents. **a** Cell viability assay on HepG2 and Huh7 cells by combined treatment with chemotherapeutic agents and AMSC-derived exosomes. **b** FITC-Annexin V/PI assay in exosome-treated HCC cells after 5-FU treatment. **c** FC analysis for Annexin V revealed an increase in apoptotic cells in 122-Exo-treated HCC cells after 5-FU or sorafenib exposure. **d**, **e** Cell cycle analysis revealed an increase in G0/G1 population in 122-Exo-treated HepG2 cells after 5-FU or sorafenib exposure. Data are presented as means ± SE. (*p < 0.05, **p < 0.01, *n* = 3). *U.T* untreated, *122-Exo* AMSC-122-derived exosomes, *67-Exo* AMSC-67-derived exosomes

To determine whether 122-Exo affects the chemosensitivity of hepatoma cells in vitro, HepG2 and Huh7 cells were exposed to chemotherapeutic agents combined with 122-Exo or 67-Exo. The growth inhibition of 5-FU or sorafenib on HCC cells was not altered by 67-Exo treatment, whereas the inhibitory effect of 5-FU or sorafenib on 122-Exo-treated HCC cells significantly increased in comparison with 67-Exo-treated control, particularly on HepG2 cells (Fig. 4a).

Flow cytometric analysis with Annexin V/PI staining also revealed an increase of apoptotic cells among 122-Exo-treated HCC cells, especially in HepG2 cells, relative to 67-Exo-treated cells (Fig. 4b, c). The apoptotic rate between 67-Exo-treated and chemotherapeutic agent only-treated HCC cells presents no difference.

Subsequently, we tested whether cell cycle arrest contributed to the enhanced growth inhibition by 122-Exo treatment. As shown in Fig. 4e, treatment of HepG2 cells with 122-Exo resulted in an increase in the percentage of G0/G1 population from 67.0 ± 1.6 % (5-FU) and 63.4 ± 1.1 % (sorafenib) to 80.4 ± 1.7 % (122-Exo and 5-FU) and 74.7 ± 3.1 % (122-Exo and sorafenib) with 5-FU or sorafenib exposure, respectively. Moreover, the treatment of Huh7 cells with 122-Exo showed similar G0/G1 arrest trends.

These data indicate that exosomes from miR-122-modified AMSCs can increase the chemosensitivity of HCC cells by enhancing cell apoptosis and cell cycle arrest.

### 122-Exo sensitizes HCC cells to sorafenib in vivo

**Fig. 5:**
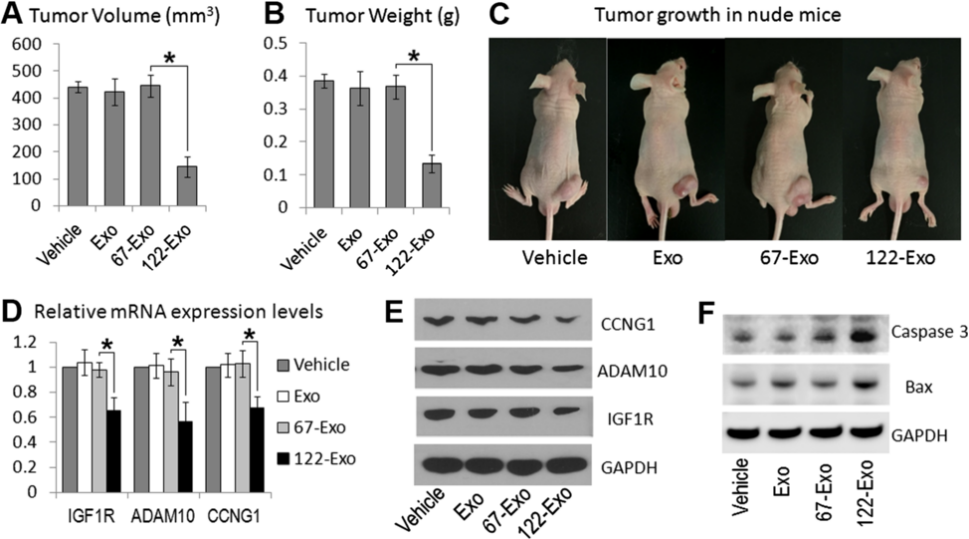
Intra-tumor injection of 122-Exo sensitizes HCC cells to sorafenib in vivo. **a**, **b** Tumor volume **a** and weight **b** measurement of HepG2 xenograft tumors at 35 days post-implantation (28 days after exosome administration combined with sorafenib treatment). **c** Imaging of mice at 35 days post-implantation. **d**, **e** Real-time PCR (**d**) and Western blot (**e**) analyses of miR-122-targeted gene expression in tumor samples at 35 days post-implantation. **f** Western blot analysis of apoptosis-related gene expression in the above tumor samples. Data are presented as means ± SE. (*p < 0.05, *n* = 5). *Exo* naïve AMSC-derived exosomes, *122-Exo* AMSC-122-derived exosomes, *67-Exo* AMSC-67-derived exosomes

**Fig. 6:**
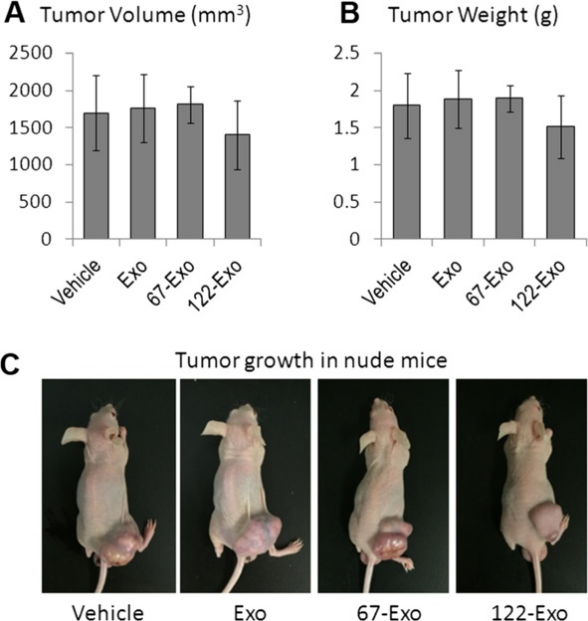
Treatment with AMSC-derived exosomes alone has no effect on HCC growth in vivo. **a**, **b** Nude mice were inoculated subcutaneously with HepG2 cells. After 7 days of tumor growth, AMSC-derived exosomes were directly administered via intra-tumor injection. Tumor volume (**a**) and weight (**b**) measurement of HepG2 xenograft tumors at 35 days post-implantation (28 days after exosome administration). **c** Imaging of mice at 28 days after sole exosome treatment. Data are presented as means ± SE (*n* = 5). *Exo* naïve AMSC-derived exosomes, *122-Exo* AMSC-122-derived exosomes, *67-Exo* AMSC-67-derived exosomes

Finally, to determine whether 122-Exo could sensitize hepatoma cells to chemotherapeutic agents in vivo, AMSC-derived exosomes (50 μg total protein in 10 μl volume) were administered to nude mice bearing HepG2 cells combined with sorafenib treatment. One intra-tumor injection of 122-Exo at 7 days after subcutaneous inoculation significantly reduced the tumor volume and weight compared with 67-Exo or vehicle-treated control. The tumor volume and weight showed no difference between naïve exosome- or vehicle-treated mice at 28 days after exosome administration and sorafenib treatment (Fig. 5a, b). Real-time polymerase chain reaction (PCR) and Western blot analysis also showed that the expression of CCNG1, IGF1R, and ADAM10 gene was downregulated in 122-Exo-treated tumors in comparison with 67-Exo-treated cells (Fig. 5d, e). The expression of apoptosis-related genes, Caspase 3 and Bax (Bcl-2 Associated X protein), was upregulated in the 122-Exo-treated group. However, treatment with AMSC-derived exosomes alone presented no effect on HCC growth. As shown in Fig. 6, no statistically significant difference was observed between exosome-treated groups and vehicle group in terms of the tumor volumes and weights at 28 days after exosome administration alone (without combination with sorafenib).

Overall, these results revealed that 122-Exo administration promotes the growth inhibitory property of sorafenib toward HCC cells.

## Discussion

Exosomes are nanometer-sized vesicles of endocytic origin that are released by multiple cell types. Exosomes essentially exert their functions as mediators of intercellular communication by transferring protein and RNA. Thus, the use of exosomes as biological delivery vehicles is of considerable interest [21, 22]. Increasing studies indicate that MSCs are well suited for the mass production of exosomes [17], which may perform important functions in the therapeutic effect of MSCs through paracrine mechanism. MSCs have been reported to potentially regulate neurite outgrowth by exosome-mediated transfer of miR-133b to neural cells [23]. Further works show that exosomes derived from miR-146-expressing MSCs can deliver miR-146 into glioma cells in vitro, as well as reduce glioma xenograft growth in a rat model of primary brain tumor [16]. The present study demonstrated a novel strategy for increasing HCC chemosensitivity through AMSCs exosome-mediated transfer of therapeutic miR-122. The miR-122-modified AMSCs can effectively package miR-122 into secreted exosomes, which mediate miR-122 communication between AMSCs and HCC cells, thus further increasing the sensitivity of HCC cells to chemotherapeutic agents through alteration of miR-122-target gene expression in these cells.

Among the predicted targets of miR-122, ADAM10, IGF1R, and CCNG1 play key roles in tumorigenesis and drug sensitivity in various cancers. ADAM10 is associated with tumor progression and confers resistance to doxorubicin-induced apoptosis in HCC cells by activation of the PI3-K/Akt pathway [24]. Signaling through IGF1R regulates HCC initiation, progression, metastasis, and resistance to therapy [25, 26]. Enhanced expression of cyclin G1 (CCNG1) contributed to drug resistance of hepatoma cells and increased recurrence rate in HCC patients [12]. In vitro experiments indicated that 122-Exo treatment resulted in the reduced expression of these genes in HCC cells over time, even at 4 weeks after122-Exo treatment (data not shown). In addition, an increase in the G0/G1-phase population and a corresponding decrease in the G2/M-phase, which is identified as a response to cyclin G1 knockdown [27], were observed in 122-Exo-treated HepG2 cells combined with chemotherapeutic agents. Therefore, by downregulating the expression levels of target genes, exosomes from miR-122-modified AMSCs can effectively increase the chemosensitivity of HepG2 cells through the induction of apoptosis and cell cycle arrest.

Given that systemic therapy with the multikinase inhibitor sorafenib is the standard of care for unresectable or advanced-stage HCC [28], we further tested whether 122-Exo can synergize its inhibitory function in mice model. A lower concentration of sorafenib (5 mg/kg) than the previously reported amount [29] was used to treat HepG2 xenograft tumors. One intra-tumor injection of 122-Exo significantly enhanced the growth inhibition by sorafenib at reduced concentration. However, the function of MSC in tumor therapy remains controversial [30]. Several studies have shown that MSC-derived naïve MVs/exosomes can inhibit tumor growth in mice models of ovarian cancer, hepatoma, multiple myeloma, and bladder tumors [31–34]. Another study reported that MSC-derived exosomes can promote vascularity and tumor growth in mice xenograft models of gastric carcinoma and colon cancer [35]. We further tested whether naïve AMSC-derived exosomes can affect tumor growth. Naive exosomes were administered into nude mice bearing HepG2 cells through one intra-tumor injection, and PBS was used as vehicle control. No significant differences were observed between the two groups in terms of tumor volume and weight at 28 days after exosome administration combined with (Fig. 5) or without sorafenib treatment (Fig. 6). These data suggest that naïve MSC-derived exosomes may not affect HCC growth and chemosensitivity by themselves. The different effect of MSC-derived exosomes on tumor growth between our study and the above studies may be ascribed to differences in tumor-bearing models, MSCs sources, and route for exosome administration. Thus, the increased sensitivity of HCC cells to sorafenib by 122-Exo administration depends on exosome-mediated miR-122 transfer and downregulation of miR-122-target genes, which was reported to be involved in the antitumor activity of sorafenib in vivo [36, 37]. However, treatment with 122-Exo alone (without combination with sorafenib) cannot significantly reduce tumor volume and weight (Fig. 6). This effect may be attributed to the possibility that without the growth retardation effect of sorafenib, one intra-tumor injection of 122-Exo was insufficient for tumor growth inhibition.

The current study directly delivered exosomes into the subcutaneous xenograft models via intra-tumor injection. A previous study demonstrated that by engineering the dendritic cells to express an exosomal membrane protein (Lamp2b) fused to αv integrin-specific iRGD peptide, natural exosomes can be used for targeted tumor therapy [38]. Additional work is needed to characterize the delivery of exosomal miRNAs in orthotopic HCC models via systemic administration. Moreover, considering that the isolation of exosomes and the culturing of MSCs include reagents and methods that are still inappropriate for human use, safety data from animal studies cannot ensure the safety of initial studies in humans. Improvement in the methods for AMSC culture and exosome purification will increase the feasibility and safety of AMSC-derived exosome therapy in clinical applications.

## Conclusions

Our data indicate that miR-122, which is delivered via AMSC exosomes, can increase the sensitivity of HCC cells to chemotherapeutic agents, thereby providing a new treatment strategy for HCC.

## Methods

### Isolation and identification of AMSCs

Subcutaneous adipose tissues were obtained from three female patients (32, 28, and 41 years old, respectively) undergoing tumescent liposuction at the First Affiliated Hospital in Hangzhou. This study was approved by the hospital’s ethics committee, and informed consent was obtained from each patients. Adipose tissue was processed as previously described [39] and maintained in MesenPRO® RS™ Medium (Gibco) containing 1 % antibiotic-antimycotic (Gibco). The phenotype profile of AMSCs (passages 3 to 6) was evaluated through flow cytometry analysis (BD Accuri® C6 flow cytometer) by using PE-labeled cluster designation 29 (CD29), CD31, CD44, CD45, CD73, CD90, CD105, and human leukocyte antigen-DR (HLA-DR) (BD Bioscience Pharmingen) antibodies [39, 40]. IgG1 was used as isotype control. The differentiation of AMSCs to chondrocytes and adipocytes was tested by using StemPro® chondrogenesis and adipogenesis differentiation kit (Gibco). Afterward, staining with Oil Red O and Alcian Blue was performed to detect adipocytes and chondrocytes, respectively [41].

### Cell culture

HepG2 cells were maintained in DMEM (Gibco) containing 10 % FBS (Gibco) and 1 % antibiotic-antimycotic.

### Plasmids and AMSC transfection

The AMSC-conditioned medium consisted of DMEM supplemented with 10 % fetal bovine serum (FBS) from which bovine exosomes and protein aggregates were removed by ultracentrifugation at 100,000 *g* and 4 °C for 16 h. Prior to transfection, 1 × 10^6^ AMSCs were seeded in 10 mL of AMSC-conditioned medium overnight. AMSCs were then transfected with plasmids of hsa-miR-122 or cel-miR-67, which contained no known mRNA-binding targets in human (GenScript) by using Lipofectamine™ 2000 (Invitrogen). At 48 h after transfection, AMSCs were harvested for real-time PCR analysis.

### Isolation and identification of AMSC-derived exosomes

After 48 h of miRNA transfection, exosomes were isolated from the AMSCs supernatant by using an ExoQuick-TC Kit (System Biosciences, CA) in accordance with the manufacturer’s instructions. These exosomes were then characterized by Western blotting analysis of exosome surface markers, such as CD9, CD63, and CD81 (Abcam). Precipitates from AMSC-conditioned media were used as negative control. The protein content of exosomes was determined by using BCA protein assay kit (Pierce). Subsequently, exosome pellets were resuspended in sterile PBS at a total protein concentration of 5 μg/μL.

### Isolation and detection of miRNA

Total RNA enriched with miRNAs was isolated from AMSCs or exosomes by using a miRVana miRNA isolation kit. Real-time PCR was then performed following the manufacturer’s instructions (Ambion Diagnostics, TX) to examine miR-122 expression. Data were normalized over the average cycle threshold (CT) value of U6, and 2^-ΔΔCT^ method was used to determine the relative miRNA expression.

### Confocal microscopy studies

AMSCs were labeled with the phospholipid membrane dye, lipophilic carbocyanine DilC_16_ (3) (1.25 μM) [42]. After 10 min of incubation at 37 °C, cells were washed and resuspended in fresh media for 48 h. Fluorescent exosomes were collected and added into recipient HepG2 cells. Afterward, cells were fixed with methanol, mounted on slides, and imaged via confocal microscopy (Olympus). Background fluorescence was subtracted using unstained cells.

### RNA isolation and real-time PCR

Total RNA was isolated from HepG2 cells or tumor samples by using TRIzol, followed by real-time PCR analysis with ABI Prism 7900 (Applied Biosystems, Foster City, CA) to examine the expression of CCNG1, ADAM10, and IGF1R. GAPDH was used as an internal control. The 2^-ΔΔCT^ method was employed to determine the relative mRNA expression.

### Western blot analysis

HepG2 cells or tumor samples were lysed with RIPA peptide lysis buffer (Beyotime Biotechnology, Jiangsu, China) containing 1 % protease inhibitors (Pierce). The protein content of different fractions was detected via BCA method. Equivalent amounts of protein (20 μg) were separated by 10 % SDS-PAGE gels and transferred to polyvinylidene difluoride membranes (Millipore, Bedford, MA) and blocked with 1 % BSA in TBST for 1 h at room temperature. The membrane was incubated with CCNG1, IGF1R, ADAM10, Bax, Caspase 3, or GAPDH (Abcam) antibodies overnight at 4 °C. After washing, the membrane was incubated with HRP-conjugated secondary antibody (1:3000; Huabio) for 1 h. Blots were visualized via ECL-associated fluorography (Millipore).

### Cell viability assay

HepG2 and Huh7 cells were plated in 96-well plates at the concentration of 2 × 10^3^/well and treated with 5-FU or sorafenib combined with or without exosomes (50 ng/μL). At 72 h after culture, cell viability was determined by an 3-(4,5-dimethyl-2-thiazolyl)-2,5-diphenyl-2-H-tetrazolium bromide (MTT) assay (Sigma).

### Flow cytometry analysis of cell apoptosis and cell cycle

Cells were plated in 6-well plates at the concentration of 2 × 10^5^/well and treated with 5-FU (10 μM) or sorafenib (5 μM) combined with or without exosomes (50 ng/μL). At 48 h after treatment, cell apoptosis and cell cycle were detected using an Annexin V/PI detection kit (BD Biosciences) and cell-cycle staining kit (MultiSciences), in accordance with the manufacturer’s instructions and then analyzed on a BD Accuri® C6 flow cytometer.

### Xenograft models and exosome treatment

Male Balb/c nude mice (6 weeks old) were purchased from Zhejiang Academy of Medical Science. All experimental procedures were conducted in accordance with the Chinese legislation regarding experimental animals. Mice were inoculated subcutaneously with HepG2 cells (5 × 10^6^). After 7 days of tumor growth, mice were randomized into eight groups prior to exosome treatment. The groups comprised four groups for sole exosome administration and another four groups for exosome administration combined with sorafenib treatment. Exo (naive MSC-derived exosomes), 67-Exo (AMSC-67-derived exosomes), or 122-Exo (AMSC-122-derived exosomes) suspension (50 μg total protein in 10 μL volume) was directly administered via intra-tumor injection into each mice (*n* = 5/group), respectively. PBS was used as vehicle control. Sorafenib (5 mg/kg) was administered by intraperitoneal (i.p.) injection for five consecutive days of each week. Tumor volumes were calculated using the following formula: tumor volume (mm^3^) = 0.5 × (*W*)^2^ × (*L*), where *L* represents the length and *W* represents the width.

### Statistical analysis

Differences between groups were analyzed by using conventional Student’s *t* test or ANOVA. Each experiment was repeated at least thrice, and data were presented as mean ± SE (standard error). A *p* value of 0.05 or less was considered as statistically significant.

## Abbreviations

122-Exo: AMSC-122-derived exosomes
67-Exo: AMSC-67-derived exosomes
ADAM10: A distintegrin and metalloprotease 10
AMSC-122: miR-122-transfected AMSCs
AMSC-67: Cel-miR-67-transfected AMSCs
AMSCs: Adipose tissue-derived MSCs
CCNG1: Cyclin G1
Exo: Naïve AMSC-derived exosomes
HCC: Hepatocellular carcinoma
i.p.: Intra-peritoneal
IGF1R: Insulin-like growth factor receptor 1
MSCs: Mesenchymal stem cells
MVs: Microvesicles
TACE: Transarterial chemoembolization
U.T: Untreated
